# Clinical Outcomes of Conservative Surgery for Diffuse Uterine Leiomyomatosis: Preliminary Experience of 17 Cases in a Single Center

**DOI:** 10.3390/jcm12247638

**Published:** 2023-12-12

**Authors:** Sojung Kweon, Joowon Park, Youngseo Sim, Jae Young Kwack, Yong-Soon Kwon

**Affiliations:** Department of Obstetrics and Gynecology, Nowon Eulji Medical Center, College of Medicine, Eulji University, Hangeulbiseok-ro 68, Seoul 01830, Republic of Korea; 20210369@eulji.ac.kr (S.K.); 20210757@eulji.ac.kr (J.P.); 20210761@eulji.ac.kr (Y.S.); kjy@eulji.ac.kr (J.Y.K.)

**Keywords:** conservative surgery, fertility, diffuse uterine leiomyomatosis

## Abstract

This study aimed to introduce the clinical outcomes of conservative surgery for diffuse uterine leiomyomatosis, which also included the specialized surgical technique. All patients with diffuse uterine leiomyomatosis underwent conservative surgery such as transient occlusion of the uterine arteries (TOUA) adenomyomectomy. All 17 surgeries were performed by a single surgeon between 2018 and 2021. The mean age of the 17 patients was 36.12 years old (range 29–48, SD = 5.4). Fourteen of the 17 patients received a previous myomectomy via a laparotomic (6, 35.3%), laparoscopic (6, 35.3%), or hysteroscopic (2, 11.8%) approach. The major symptom was menorrhagia (94.1%); the mean operation time was 97.06 min (70–160, SD = 22.71), and the mean estimated blood loss was 283.53 mL (20–1000, SD = 273.72). The mean hemoglobin level one day after the operation was 9.64 g/dL (7.2–13.1, SD = 1.85). The mean hospital stay was 6.47 days (6–8, SD = 0.62). The mean follow-up duration was 116.41 weeks (32–216, SD = 50.88). The recurrence rate was 5/17 (29.4%), and the recurrence-free interval was 50.6 weeks (27–87, SD = 23.71). In patients with diffuse uterine leiomyomatosis, who want fertility preservation and relief of disease-related symptoms, conservative surgery such as TOUA adenomyomectomy could be a good option to preserve the uterus. However, further studies are required to assess fertility outcomes with a long-term follow-up.

## 1. Introduction

Leiomyomas are the most common benign uterine neoplasm [1]. They are encountered in up to 25% of women in active reproductive life [2,3]. Leiomyomatosis usually presents either as a single nodular mass or as a condition with multiple nodules distributed throughout all the parts of the uterus. Diffuse uterine leiomyomatosis is a rare condition in which the uterus is symmetrically enlarged because of the almost complete replacement of the myometrium by innumerable, poorly defined, confluent nodules [4,5,6]. The diameter of each myoma in diffuse leiomyomatosis is small (all having a diameter that usually ranges from 0.5 to 3.0 cm), with myomas distributed throughout all the areas of the uterus except the cervix. While usual leiomyomas are well confined masses with an asymmetrical involvement of the uterus, most of those related to diffuse leiomyomatosis have indistinct margins and are combined together [1,7]. Therefore, complete excision of all masses and preservation of the uterus is difficult [8,9,10].

Similar to those with uterine leiomyomas, patients with diffuse leiomyomatosis present with menorrhagia, dysmenorrhea, abdominal pain, infertility, and pelvic pressure [4]. Clinically, it presents in women between the third and fourth decades of life [2,5]. While hormonal treatment effectively controls the tumor growth and symptoms, it usually fails to control the symptoms, anemia, or tumor growth after treatment cessation [11]. Furthermore, suppression of ovarian estrogen production with a superactive agonistic analog of gonadotropin-releasing hormone is associated with a decrease in uterine myoma size, but myomas may regrow with reinstitution of ovarian function [12].

Conventionally, the standard surgery of choice for diffuse uterine leiomyomatosis has been hysterectomy, as hormonal medical treatment often fails to control the symptoms. However, as the majority of patients with diffuse uterine leiomyomaotosis are of reproductive age, hysterectomy is a difficult choice unlikely to be easily accepted by women who want to save fertility for a future pregnancy [13,14]. In addition, profuse bleeding during surgery is an obstacle to conservative surgery.

Further medication, uterine artery embolization (UAE), and hysteroscopic myomectomy have shown partial effectiveness; however, hysterectomy is currently the only known treatment capable of eliminating the symptoms of leiomyomatosis. Consequently, hysterectomy has been the only permanent treatment option for all patients, including those in the third and fourth decades, for symptoms related to uterine fibroids in diffuse leiomyomatosis [9].

However, patients with reproductive desires require alternative treatment strategies that can preserve uterine function and fertility. With the current trend of increased age at marriage, the mean age for women getting married has increased. Therefore, there is a need for better treatment modalities to eliminate leiomyomatosis and retain uterine function.

In this study, we designed a uterus-conserving leiomyomatosis treatment method that surgically eradicates most lesions and preserves the uterus. Furthermore, we evaluated the safety and efficacy of conservative surgical treatment in patients with diffuse uterine leiomyomatosis performed by a single surgeon at Eulji University Hospital in Seoul, South Korea.

## 2. Materials and Methods

**Figure 1:**
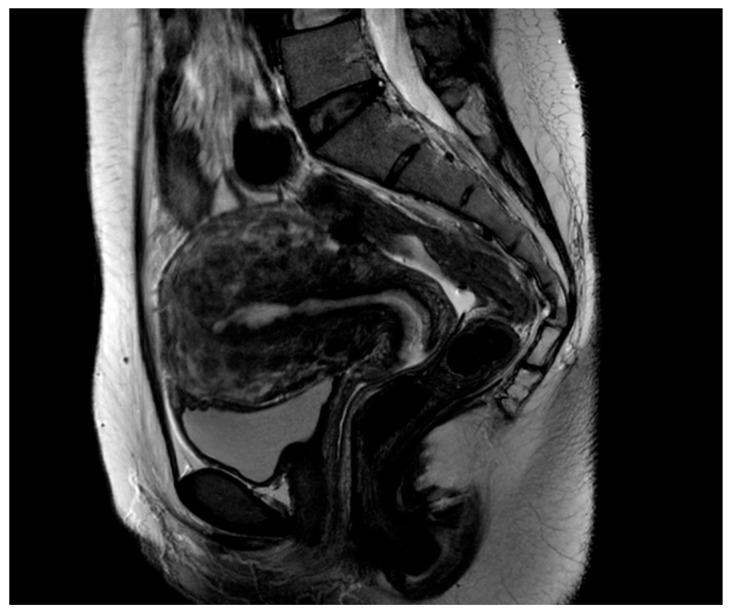
Sagittal T2-weighted magnetic resonance (MR) image shows multiple nodules with low-signal intensity in the whole uterus. An enlarged uterus shows diffuse innumerable submucosal, interstitial, and subserosal fibroids, with innumerable small fibroids blending and totally replacing the uterine parenchyma.

From August 2018 to April 2021, patients with symptomatic diffuse uterine leiomyomatosis refractory to conservative medical treatment, and who had a strong desire for the preservation of the uterus, were enrolled in this study. We performed conservative surgery, such as transient occlusion of the uterine arteries (TOUA) adenomyomectomy, via a laparotomic approach in 17 patients with leiomyomatosis. The patients had been clinically diagnosed preoperatively based on findings from magnetic resonance imaging or vaginal sonography, and showed a symmetrically enlarged uterus and numerous small nodules that displaced the uterus and endometrium [15] (Figure 1). Patients received a single-cycle gonadotropin-releasing hormone agonist (GnRHa) two weeks before the operation date to induce a thin stabilized endometrium.

The weight of the diffuse leiomyomatosis lesion was defined as the total weight of the excised lesion. The operating time was defined as the period from skin incision to closure, and operative blood loss was estimated by subtracting the rinse volume from the blood volume collected in the suction apparatus. Three cycles of adjuvant GnRHa were injected subcutaneously at monthly intervals postoperatively. At the 7 month follow-up, we assessed the improvement in symptoms, including dysmenorrhea and menorrhagia, using questionnaires. The questionnaire focused on evaluating the presence and severity of dysmenorrhea and menorrhagia. This was performed through a simple clinical interview. An 11-point numerical rating scale was used to evaluated the intensity of pain during menstruation (0 = no pain to 10 = excruciating pain) [16]. The Mansfield–Voda–Jorgensen (MVJ) menstrual bleeding scale, used to evaluate menorrhagia, is a subjective Likert-type scale from 1 (spotting) to 6 (very heavy bleeding or gushing) [17]. Complete remission of dysmenorrhea was defined as 0 on the numerical rating scale, and complete remission of menorrhagia was defined as 0 on the MVJ scale at 8 months after the procedure. Regular follow-ups at 6 month intervals without any medications was performed at the outpatient clinic using ultrasound.

SPSS software (version 22.0; IBM Corp., Armonk, NY, USA) was used for the statistical analysis. Results are expressed as mean ± standard deviation (SD) or absolute numbers.

### Surgical Technique

General anesthesia was induced, and the patient was placed in a supine position with endotracheal intubation. We performed bilateral isolation and occlusion of the uterine arteries with a vascular clip throughout the surgery. We named this procedure transient occlusion of uterine arteries (TOUA). Using a blunt-tip suction, the branching uterine artery along the umbilical artery was isolated. The isolated uterine artery was clipped temporally, and vascular clips were removed once uterine reconstruction was completed.

**Figure 2:**
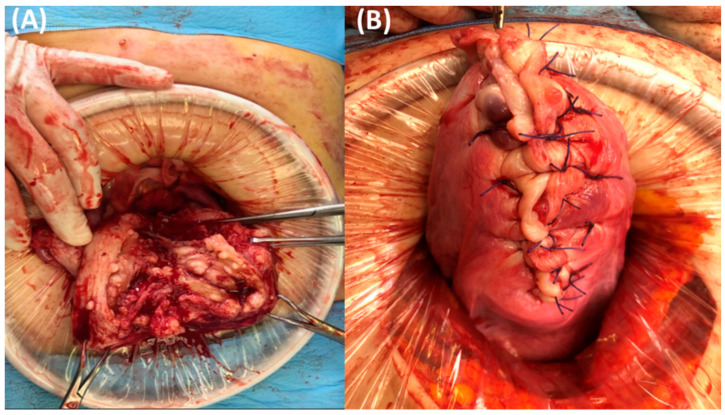
(**A**) Appearance of the longitudinally-dissected uterus, cut along the center of the uterine cavity. Countless diffuse multiple submucosal and intramural nodules are located in the myometrium of the uterus. (**B**) Final picture when the uterus was completely repaired.

For excising the diffuse leiomyomatosis lesion, the uterus was dissected perpendicular to the axis using a scalpel, and the endometrial cavity at the uterine fundus was exposed (Figure 2).

**Figure 3:**
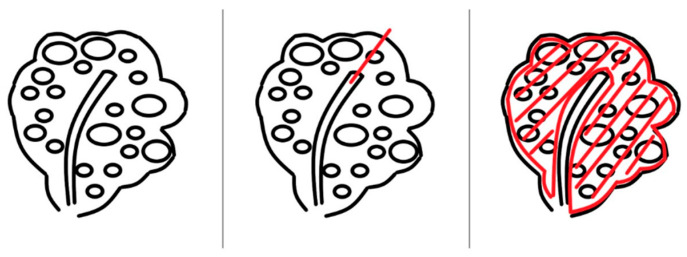
Description of the excision of diffuse leiomyomatosis. First, an excision was made from the endometrium. Then, the myometrial space was cut through the endometrial line from the outer edge of the endometrium with the scalpel 5 mm away. To separate the endometrial cavity and myometrium, a deep incision was made. Finally, an incision line was made from the serosal space. The excision procedure was performed at the area developed by the two incisions. A complete myomectomy was performed using this procedure.

From the incision site, each side was laterally divided into two parts. Multiple myomas involving the entire myometrium were exposed. Excision was started approximately 5 mm from the endometrium to preserve the endometrial architecture using a scalpel with the surgeon’s finger tactile sensation. A deep incision was made to separate the endometrial tissue from the myometrium. The next step was to make the incision line approximately 3 mm from the uterine serosal layer. The excision procedure was performed in the area developed by two incisions (Figure 3).

**Figure 4:**
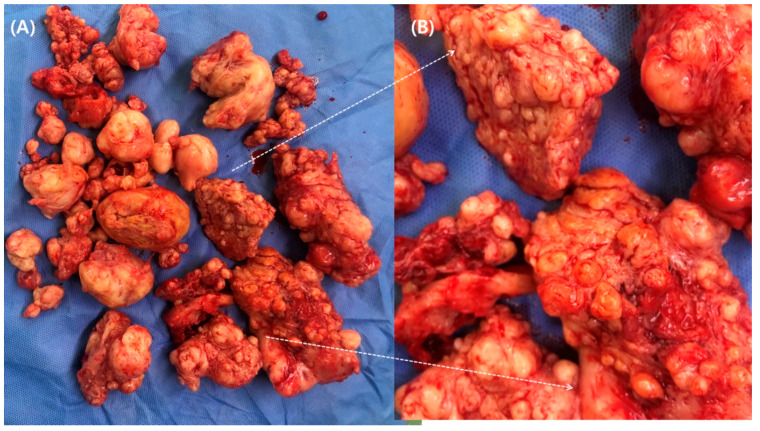
(**A**) Specimen of radical myomectomy. (**B**) A magnified picture shows diffuse leiomyomatosis with surrounding myometrium.

Numerous myomas with surrounding myometrial tissues were removed (Figure 4). Most of the myometrium was excised, except for the region consisting of the endometrial cavity and serosa, which is needed to reconstruct the shape of the uterus.

After excision, the endometrial lining was closed using a continuous, running suture with a 3-0 multifilament synthetic absorbable suture. The spaces created by complete excision of the diffuse leiomyomatosis lesions were sutured and closed with simple interrupted sutures of 2-0 Polysorb (^®^COVIDIEN, 710 Medtronic Pkwy US Minnesota Fridley), starting from the lower and lateral side and then in the central and upward excision line. The same procedure was performed on each side of the left, right anterior, and posterior walls of the uterus. The final closure of the uterine serosa layers was performed layer by layer.

After uterine reconstruction, two vascular clips occluding the uterine arteries were removed. Thorough irrigation with warm saline for approximately 2–3 min was performed to detect delayed bleeding. When the peritoneum was closed, a drain was inserted into the pelvic cavity. The abdominal incision was closed in layers.

## 3. Results

The mean age of the 17 patients was 36.12 years old (29–48, SD = 5.4). Fourteen of the 17 patients underwent previous myomectomy via a laparotomic (6, 35.3%), laparoscopic (6, 35.3%), or hysteroscopic (2, 11.8%) approach for symptom relief. The major symptom was menorrhagia (94.1%). Eleven patients had never been pregnant, five had experienced miscarriages, and two had experienced deliveries (Table 1).

The mean operation time was 97.06 min (70–160, SD = 22.71), and the mean estimated blood loss was 283.53 mL (20–1000, SD = 273.72). One patient required an intraoperative transfusion due to heavy bleeding. In all of the cases, the pathology reports confirmed diffuse leiomyomatosis. The mean total weight of the enucleated myomas was 345.41 g (89–910, SD = 240.65). There were no cases of uterine arteries or pelvic nerve injuries. The conversion rate to hysterectomy was zero. The mean hemoglobin level one day after the operation was 9.64 g/dL (7.2–13.1, SD = 1.85), and the mean hospital stay was 6.47 days (6–8, SD = 0.62). All patients were discharged without serious postoperative complications (Table 2).

Symptom relief was 100% without any medication after completion of the GnRHa injection. The mean follow-up duration was 116.41 weeks (32–216, SD = 50.88). The recurrence rate was 5/17 (29.4%), and the recurrence-free interval time was 50.6 weeks (27–87, SD = 23.71). One patient had taken dienogest, a kind of oral progestin, because she wanted to stop menstruation continuously due to excessive worry about heavy bleeding again despite receiving the surgery; however, no medication to relieve serious symptoms was prescribed (Table 3). There were no cases of reoperation in patients with recurrence.

## 4. Discussion

Unlike a typical uterine leiomyoma, a diffuse uterine leiomyomatosis is a severe form in which most of the uterine parenchyma is replaced with countless leiomyomas. Although uterine leiomyoma has a wide range of treatment options, the favored treatment for leiomyoma-related symptoms (i.e., menorrhagia, intermenstrual or menstrual pelvic pain, and increased urinary frequency or bloating summarized as bulk-related symptoms) is hysterectomy [12,18].

There have been few reports of conservative treatments other than surgery for diffuse leiomyomatosis, including hormone therapy, UAE, and transcervical resection [19]. Koh et al. reported that UAE is a highly effective treatment for diffuse uterine leiomyomatosis with mid-term durability, and may be a valuable alternative to hysterectomy [20]. However, some physicians refrain from recommending UAE to women over 40 years of age, as it has been shown to negatively affect fertility [21,22]. One study’s results are concerning, as they show loss of ovarian reserve in a population of women with a mean age of 44 years following UAE [23]. The patient’s age is often high, close to 40–44 years, with possible natural disturbance of menses [24]. The hypothesis found in the literature is that UAE could precipitate menopause in women aged over 40 years or premenopausal women [25,26,27]. Therefore, the use of UAE is limited when managing women who want to preserve their fertility. Conclusively, the effectiveness of previous conservative treatment for diffuse uterine leiomyomatosis could not be proven in women who desire the preservation of fertility.

In addition, partial myomectomy via any approach was not effective in managing the patients with symptomatic diffuse uterine leiomyomatosis, because the remaining lesions can easily relapse and regrow. In this study, 14 of the 17 patients (82.4%) had a history of partial myomectomy, and the previous surgery could not provide a symptom-free time without medication, requiring another conservative surgery afterward.

The purpose of postoperative GnRH agonist treatment was not to treat residual lesions, but to use it to aid in the regeneration process. There were two reasons for the administration of GnRHa injections that are similar to the background of TOUA adenomyomectomy. GnRHa induces hypoestrogenism, which shrinks fibroids and the endometrium [28]. In our study, GnRHa was injected once two weeks before the operation, and three cycles of GnRHa injections were administered after the operation. The thin line and stability of the endometrium during operation are required to perform a fine approximation of the two incised endometrium and obtain a normal architectural endometrium, which can be made by a single administration of GnRHa injection before the operation. The purpose of postoperative GnRH agonist treatment was not to treat residual leiomyomatosis lesion, but to aid in the regeneration process of the uterus.

Three postoperative cycles of GnRHa injections were required to reduce or block the menstrual flow of blood and endometrial tissues from the operation-related healing site of the uterus.

In diffuse leiomyomatosis, conventional myomectomy using an external approach is challenging for surgeons as a fertility-sparing procedure [29]. Hysteroscopy is the standard treatment for submucosal leiomyomas that undergo intracavitary development; however, the effectiveness of hysteroscopic surgery in diffuse uterine leiomyomatosis is limited [19].

Nishida et al. previously demonstrated conservative myomectomy in seven cases of leiomyomatosis [19]. The incision method was similar to that in our study; however, myomas were enucleated without excision of the surrounding myometrium. Their method could remove more myomas than conventional myomectomy; however, complete resection seemed difficult to achieve. The operation time was long (mean 284 min), and the volume of blood loss (mean 1614 g) was large, with three patients requiring blood transfusion. As this study shows, operative time and blood loss are strongly correlated. This means that shortening the operative time is extremely useful for reducing blood loss. To reduce intraoperative bleeding we used the TOUA procedure, which has been used in several gynecologic conditions. Based on our results, the TOUA technique could minimize intraoperative blood loss.

**Figure 5:**
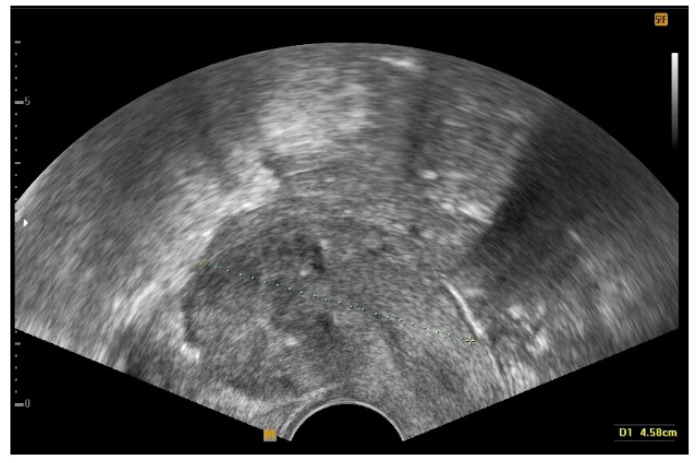
Transvaginal ultrasonography of the uterus during follow-up after the conservative surgery. The image shows a complete eradication of diffuse leiomyomatosis.

Additionally, TOUA can stabilize the vital signs of patients during surgery and permit the handling of a prolonged and fine procedure without heavy intraoperative bleeding loss. This study showed that TOUA is a useful surgical tip for safely performing operations with expected massive bleeding such as conservative surgery, because estimated blood loss and operation time might be comparable. Furthermore, the benefit of TOUA is the normalization of uterine arterial flow right after the surgery. In TOUA, uterine arteries are clipped temporarily, which does not damage the uterine blood supply related to ovarian and endometrial function. It has been reported that the permanent occlusion of uterine arterial flow can lead to endometrial and ovarian dysfunction that could induce subfertility [23,30,31,32]. Permanent occlusion of uterine arteries is a similar procedure to uterine artery embolization, and is thought to be one of the causes of lowering the pregnancy rate, so that transient clamping of uterine arteries was performed to prevent bleeding during the surgery. Unlike myoma enucleation, the involved myometrium was excised with myoma enucleation in this study, which might allow for a near-complete excision of the lesion and successful uteroplasty (Figure 5).

In the present study, we performed a surgical therapy conservative surgery for diffuse uterine leiomyomatosis such as TOUA adenomyomectomy, which involves the excision of the myoma and surrounding myometrium to remove as much myoma as possible. It is distinct from a conventional myomectomy, which involves the enucleation of each myoma nodule. During surgery, we noticed that most of the myometrium consisted of interconnected myomas; therefore, we changed the concept of myomectomy from enucleation to complete resection. Although five patients showed a remnant or recurrent leiomyoma that was more than 1.0 cm in maximum diameter as shown by ultrasonography, they did not have recurrent menstruation-related symptoms.

This study demonstrates that our technique is a potential and useful option for conservative treatment; however, it is limited in that it is a single-arm study with a short follow-up period and a small number of enrolled patients. To assess the fertility and delivery outcomes of radical myomectomy in this study, long-term follow-up will be needed, and more patients should be enrolled in future studies. As it is expected to take a few more years, a follow-up study about pregnancy and delivery outcome after the conservative surgery will be reported in the near future. Another limitation is that it is difficult to adjust for the skills and differences in technique among individual surgeons. In this study, operator bias was minimized by employing one surgeon.

Several studies have dealt with a conservative management of diffuse leiomyomatosis. We compared the number of patients treated using various conservative management strategies. Most of the studies were case reports with only one case enrolled. Five of these 11 studies were case reports. Six studies included more than two cases (Range 3–8) (Table 4). Our study shows 17 compatible patients compared to previously published studies. This indicates that a conservative treatment method is by far the most credible. In addition, through this study, we could set guidelines for women with diffuse leiomyomatosis who want to preserve their fertility.

## 5. Conclusions

In summary, this retrospective analysis of 17 patients with symptomatic diffuse uterine leiomyomatosis demonstrated that conservative surgery for diffuse uterine leiomyomatosis, such as TOUA adenomyomectomy, leads to marked and sustained improvement in clinical symptoms, and is not associated with periprocedural complications. For relief of leiomyomatosis-related symptoms in patients with diffuse uterine leiomyomatosis involving the whole uterus, conservative surgery of diffuse uterine leiomyomatosis such as TOUA adenomyomectomy could be a surgical treatment option to preserve the uterus. However, it is limited in that it is a single-arm study with a low number of patients, and the follow-up period is too short. Therefore, future assessments will be performed to assess long-term outcomes, including the recurrence of symptoms and leiomyomatic lesions, and fertility outcomes.

## Figures and Tables

**Table 1:** Baseline characteristics of conservative surgery for diffuse uterine leiomyomatosis (N = 17).

Mean age (years)	36.12 (29–48, SD = 5.4)
Previous myomectomy	
Laparotomic	6 (35.3%)
Laparoscopic	6 (35.3%)
Hysteroscopic	2 (11.8%)
None	3 (17.6%)
Marital status	
No	8 (47.1%)
Yes	9 (52.9%)
Abortion	
No	4/9 (44.4%)
Yes	5/9 (55.6%)
Main symptom	
Menorrhagia ^a^	16 (94.1%)
Dysmenorrhea ^b^	0
Combined	1 (5.9%)

SD: standard deviation. ^a^. Mansfield–Voda–Jorgensen (MVJ) menstrual bleeding scale was used for assessment. Complete remission of menorrhagia was defined as 2–3 on the MVJ scale, and partial remission was defined as decreased score more than 50% improvement in symptoms at 7 months after the procedure. ^b^. Dysmenorrhea was assessed by numerical rating scale score during the menstrual period. Complete remission of dysmenorrhea was defined as 0 on the numerical rating scale, and partial remission was defined as 2–3 on the numerical rating scale at 7 months after treatment.

**Table 2:** Surgical outcomes of conservative surgery for diffuse uterine leiomyomatosis (N = 17).

Operation time (minute)	97.06 (70–160, SD = 22.71)
EBL (mL)	283.53 (20–1000, SD = 273.72)
Weight of excised specimen (gram)	345.41 (89–910, SD = 240.65)
Intraoperative transfusion (N = 17)	
No	16 (94.1%)
Yes	1 (5.9%)
Hb 2 week before operation	12.32 (10–14.9, SD = 1.57)
Hb 1 day after operation	9.64 (7.2–13.1, SD = 1.85)
Hospital stay (day)	6.47 (6–8, SD = 0.62)

EBL (estimated blood loss). Hb (g/dL): hemoglobin after or before surgery. SD: standard deviation.

**Table 3:** Recurrence outcomes of conservative surgery for diffuse uterine leiomyomatosis (N = 17).

Mean follow-up duration (weeks)	116.41 (32–216, SD = 50.88)
Relief of symptoms recurrence ^a^	100%
No	12 (70.6%)
Yes	5 (29.4%)
Recurrence-free interval (weeks)	50.6 (27–87, SD = 23.71)
Medication for recurrence (N = 5)	
No	4 (80%)
Yes	1 (20%)
Reoperation	0 (0%)

^a^. Recurrence: new lesion size up to above 1 cm with relapsed symptoms during follow-up period with a 6 month interval. Five patients showed a remnant or recurrent leiomyoma which was more than 1.0 cm in the maximum diameter as shown by ultrasonography, but they did not have menstrual related symptoms that they had previously.

**Table 4:** Cases of conservative treatment in diffuse leiomyomatosis patients.

Case	Reference	Study	Enrolled Number	Symptoms	Treatment	Outcome
1	Jieun Koh et al. [20]	Retrospective	7	Menorrhagia	Uterine artery embolization	Symptom relief
2	Li Chen et al. [33]	Case report	1	Menorrhagia Dysmenorrhea	High intensity focused ultrasound	Symptom control
3	Xiaofei Zhang et al. [34]	Research article	8	Menorrhagia Dysmenorrhea	High intensity focused ultrasound	Uterine volume shrinkage
4	Luigi Fedele et al. [35]	Descriptive study	3	Menorrhagia	Myomectomy	Menstrual pattern, pregnancy
5	Yasuo Otsubo et al. [5]	Case report	1	infertility	Myomectomy	Pregnancy
6	Masato Nishida et al. [19]	Original article	7	Menorrhagia	Myomectomy	Anemia improved. Pregnancy
7	Christian Scheurig et al. [18]	Case report	6	Menorrhagia Dysmenorrhea	Uterine artery embolization	Alleviation of symptoms
8	Aki Kido et al. [36]	Case report	1	Hypermenorrhea Dysmenorrhea	Uterine artery embolization	Reduced volume
9	Sudhir Kunchala et al. [37]	Case report	1	Menorrhagia Dysmenorrhea	Uterine artery embolization	Resolution of symptoms
10	Yuji Hiramatsu et al. [38]	Case report	3	Menorrhagia	Myomectomy	
11	Ikuo Konishi et al. [39]	Case report	1	Menorrhagia Dysmenorrhea Infertility	Myomectomy	Pregnancy
12	Sojung Kweon et al. (this work)	Original article	17	Menorrhagia Dysmenorrhea	Myomectomy	Symptoms relief Reduced volume

## Data Availability

The datasets used and/or analyzed during the current study are available from the corresponding author on reasonable request.
